# New aspects on efficient anticoagulation and antiplatelet strategies in sheep

**DOI:** 10.1186/1746-6148-9-192

**Published:** 2013-10-03

**Authors:** Annika Weigand, Anja M Boos, Jürgen Ringwald, Maren Mieth, Ulrich Kneser, Andreas Arkudas, Oliver Bleiziffer, Dorothee Klumpp, Raymund E Horch, Justus P Beier

**Affiliations:** 1 Department of Plastic and Hand Surgery, University Hospital of Erlangen Friedrich-Alexander University of Erlangen-Nürnberg, Krankenhausstr. 12, Erlangen D-91054, Germany; 2 Department of Transfusion Medicine and Hemostaseology, University Hospital of Erlangen, Friedrich-Alexander University of Erlangen-Nürnberg, Erlangen, Germany; 3 Institute of Experimental and Clinical Pharmacology and Toxicology, Friedrich-Alexander University of Erlangen-Nürnberg, Erlangen, Germany; 4 Department of Hand, Plastic and Reconstructive Surgery, Burn Center, BG Trauma Center, Ludwigshafen, Germany

**Keywords:** Acetylsalicylic acid, Aggregometry, Anticoagulation therapy, Clopidogrel, Dabigatran etexilate, Factor Xa inhibitor, Platelet aggregation inhibitor, Sheep model, Sodium enoxaparin, Ticagrelor

## Abstract

**Background:**

After addressing fundamental questions in preclinical models *in vitro* or in small animals *in vivo,* the translation into large animal models has become a prerequisite before transferring new findings to human medicine. Especially in cardiovascular, orthopaedic and reconstructive surgery, the sheep is an important *in vivo* model for testing innovative therapies or medical devices prior to clinical application. For a wide variety of sheep model based research projects, an optimal anticoagulation and antiplatelet therapy is mandatory. However, no standardised scheme for this model has been developed so far. Thus the efficacy of antiplatelet (acetylsalicylic acid, clopidogrel, ticagrelor) and anticoagulant (sodium enoxaparin, dabigatran etexilate) strategies was evaluated through aggregometry, anti-factor Xa activity and plasma thrombin inhibitor levels in sheep of different ages.

**Results:**

Responses to antiplatelet and anticoagulant drugs in different concentrations were studied in the sheep. First, a baseline for the measurement of platelet aggregation was assessed in 20 sheep. The effectiveness of 225 mg clopidogrel twice daily (bid) in 2/5 sheep and 150 mg bid in 3/5 lambs could be demonstrated, while clopidogrel and its metabolite carboxylic acid were detected in every plasma sample. High dose ticagrelor (375 mg bid) resulted in sufficient inhibition of platelet aggregation in 1/5 sheep, while acetylsalicylic acid did not show any antiplatelet effect*.* Therapeutic anti-factor Xa levels were achieved with age-dependent dosages of sodium enoxaparin (sheep 3 mg/kg bid, lambs 5 mg/kg bid). Administration of dabigatran etexilate resulted in plasma concentrations similar to human ranges in 2/5 sheep, despite receiving quadruple dosages (600 mg bid).

**Conclusion:**

High dosages of clopidogrel inhibited platelet aggregation merely in a low number of sheep despite sufficient absorption. Ticagrelor and acetylsalicylic acid cannot be recommended for platelet inhibition in sheep. Efficient anticoagulation can be ensured using sodium enoxaparin rather than dabigatran etexilate in age-dependent dosages. The findings of this study significantly contribute to the improvement of a safe and reliable prophylaxis for thromboembolic events in sheep. Applying these results in future translational experimental studies may help to avoid early dropouts due to thromboembolic events and associated unnecessary high animal numbers.

## Background

Prior to clinical application, the safety and efficacy of newly developed therapies or innovative medical devices have to be ensured. Thus animal models have become an essential part of researchers' armamentarium in nearly all fields of experimental research [1]. Especially in the field of translational and preclinical research, in regard to the transferability of new therapies to humans, large animals are indispensable [2]. The sheep is a commonly used experimental animal model [3], above all due to its human similarity regarding the vascular and skeletal system. It has become an irreplaceable animal model, particularly in research concerning the cardiovascular system, as well as the investigations of heart assist devices and heart valve replacement therapy [4]. The sheep is ideally suited to investigate issues *in vitro*[5] and *in vivo*, for example in orthopaedic traumatology [6] and reconstructive surgery, especially in the field of tissue engineering [7,8], dealing with the generation of autonomously vascularised tissue. In these special experimental setups, optimal anticoagulation schemes with suitable monitoring are required to ensure successful procedures, thus avoiding early dropouts because of vascular obliteration. Studies on anticoagulation and antiplatelet therapy in sheep are very rare. Most studies were performed several years to decades ago and report small experimental groups with conflicting data on possible strategies, mostly using anticoagulation protocols already established in humans [9]. To date, the knowledge of the differences between the sheep and human anticoagulation system is limited. Only few and sometimes inconsistent data exist. For example, remarkably different concentrations of clotting factors in sheep compared to humans are reported in literature [10]. Due to the different platelet aggregation characteristics of sheep in comparison to human platelets, a reliable effect in sheep cannot be guaranteed when applying human antiplatelet therapy protocols [11,12].

In the human clinical setting, there are a variety of medical options for the prevention of thromboembolic events. In addition to the newly developed ticagrelor [13], acetylsalicylic acid (ASA) and clopidogrel are the most commonly used platelet aggregation inhibitors [14]. Anticoagulant protocols based on heparin are applied in a large number of experimental studies using the sheep *in vivo* model, however, due to short effect duration, administration every 4 hours is necessary [15]. The efficacy of the inhibition of factor Xa (FXa) by low-molecular-weight heparin (LMWH) was investigated in a few studies in adult sheep [16,17], however, never in lambs. Dabigatran etexilate is a newly developed oral direct thrombin inhibitor [18], for which the feasibility and safety of switching from enoxaparin is described in literature [19]. However, no data exist about the usage of this new thrombin inhibitor in sheep.

Since there are no optimal methods for the prevention of thromboembolic events in sheep, nor standardised measurement methods described so far for the determination of the antiplatelet effect, the aim of this study was to establish an optimal thrombosis prophylaxis strategy, as well as a testing system to ensure the efficacy of an antiplatelet and anticoagulation therapy in sheep.

## Results

### Establishment of a method for the assessment of platelet function in sheep

No standardised measurement method for the assessment of platelet function in sheep has been described so far, thus a testing system to evaluate the efficacy of antiplatelet drugs in sheep was established.

In the impedance aggregometry internal laboratory agonist concentrations for human blood were used to induce platelet aggregation in sheep blood. Adenosine diphosphate (ADP) (final concentration 6.5 μM) induced platelet aggregation to an extent expected of that for human blood (area under the curve (AUC) 738 ± 125.87 AU*min). In contrast, arachidonic acid (AA) (final concentration 0.5 mM) and thrombin receptor-activating peptide 6 (TRAP-6) (final concentration 32 μM) could not sufficiently induce platelet aggregation in sheep blood. Increasing the concentration of AA up to 6.56 mM and TRAP-6 up to 320 μM did not result in induction of platelet aggregation. Internal laboratory human reference ranges were used as target values (AUC (ADP): 534–1220 AU*min, AUC (AA): 745–1361 AU*min, AUC (TRAP-6): 941–1563 AU*min). Since not all agonists could sufficiently induce platelet aggregation, the continued use of impedance aggregometry was rejected.

In the LTA, final concentrations of 0.19 mg/mL of collagen (col) and 2 × 10^-5^ M of ADP (concentrations used for human platelet rich plasma [PRP]) induced adequate platelet final aggregation (FA) in sheep PRP. The initial AA concentration of 1.64 mM (concentration used for human PRP) had to be increased to 10.93 mM to induce platelet aggregation sufficiently.

Therefore, these concentrations were used for the determination of reference values evaluating the platelet aggregation of 20 healthy and untreated sheep (col induced FA (col-FA): 81.6 ± 14.2%, ADP-FA: 87.4 ± 11.6%, AA-FA: 81.9 ± 10.7%). Calculated cut-off values include 53.1% for col-FA, 64.2% for ADP-FA and 60.6% for AA-FA.

All sheep and lambs survived the experiments unscathed. No side effects, such as signs of haemorrhage were observed during the study. Periodically performed counting of platelets resulted in a mean platelet value of 424 ± 128 × 10^3^/μL.

The results of the experimental groups are mentioned below.

### ASA did not inhibit sheep platelet aggregation

The efficacy of ASA in sheep was tested in two different modes of application over 7 days in the LTA and through bleeding time (BT) on baseline, days 1, 4 and 7. In addition, the efficacy of a high concentration of ASA was evaluated *in vitro* using LTA.

**Figure 1:**
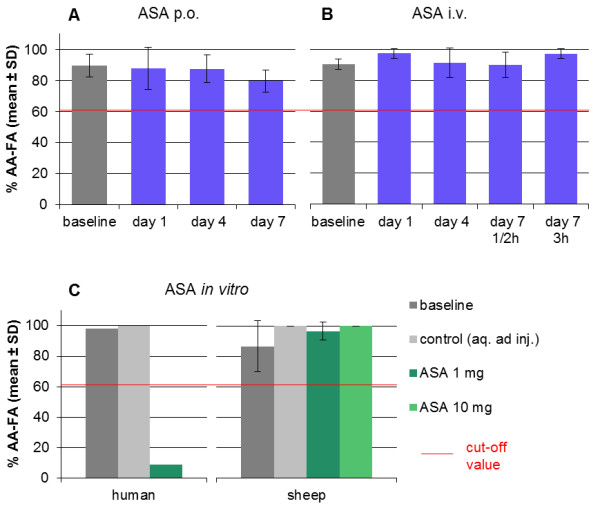
**Acetylsalicylic acid could not inhibit platelet aggregation *in vivo *and *in vitro *(Groups 1a–c). A–B**: Acetylsalicylic acid (ASA) in a concentration of 500 mg bid given per os (p.o.) in Group 1a **(A)** and intravenously (i.v.) in Group 1b **(B)** had no significant effect on arachidonic acid induced platelet final aggregation (AA-FA) in the light transmission aggregometry (LTA) between baseline and days 1, 4 and 7, respectively (one-way repeated measures ANOVA). Values did not decline below the cut-off value at any time point (red line, 60.6%). Platelet aggregation was induced by 10.93 mM arachidonic acid. **C**: *In vitro* acetylsalicylic acid failed to inhibit arachidonic acid induced platelet final aggregation of sheep platelet rich plasma. The platelet aggregation in human platelet rich plasma was inhibited with acetylsalicylic acid in a concentration of 1 mg/mL in LTA. However, no significant change in sheep platelet final aggregation could be detected after addition of 1 mg/mL or 10 mg/mL acetylsalicylic acid compared to baseline or control (Friedman Test). Values did not decline below the cut-off value at any time point (red line, 60.6%). Platelet aggregation was induced by 10.93 mM arachidonic acid. As control, aqua ad injectabilia instead of acetylsalicylic acid solution was used.

The application of 500 mg ASA twice a day (bid) per os (p.o.) (Group 1a) induced no significant differences in AA and collagen induced platelet final aggregation between baseline, days 1, 4 and 7 (AA-FA, Figure 1A).

No significant differences were induced with the application of 500 mg ASA bid intravenously (i.v.) (Group 1b) between the measurement time points during the 7-day treatment (AA-FA, Figure 1B). Final aggregation values in Groups 1a and b did not decline below the cut-off values at any time point.

BT did not change significantly either after p.o. or after i.v. administration.

The effect of ASA on sheep platelets was measured *in vitro* (Group 1c). The addition of ASA in a final concentration of 1 mg/mL and 10 mg/mL could not induce any inhibition of sheep platelet final aggregation after stimulating with AA or collagen compared to the values of the baseline and control (AA-FA, Figure 1C). Final aggregation values did not decline below the cut-off values at any ASA concentration. The effect of 1 mg/mL ASA on human platelets served as positive control (AA-FA: 9%, col-FA: 100%). After the addition of 10 mg/mL ASA, collagen induced platelet final aggregation still was 78%.

### Effect of clopidogrel on sheep and lamb platelet aggregation

The inhibition of platelet aggregation was evaluated in sheep in three different dosages over 7 days and in one dosage in lambs over 28 days using LTA and BT on baseline, days 1, 4, 7 and 28 (lambs), respectively.

No significant antiplatelet effect could be observed when clopidogrel was administered to sheep over 7 days in a concentration of 150 mg bid (Group 2a), 225 mg bid (Group 2b), 375 mg bid (Group 2c).

The application of 150 mg clopidogrel bid to lambs over 28 days (Group 2d) induced no significant differences between baseline and days 1, 4, 7 and 28.

**Figure 2:**
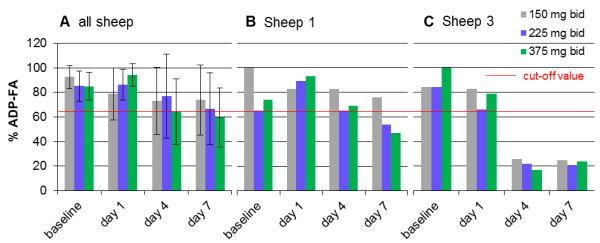
**Clopidogrel inhibited platelet final aggregation adequately in 2 out of 5 sheep (Group 2). A**: Calculated light transmission aggregometry (LTA) results of all sheep receiving clopidogrel per os in a dosage of 150 mg (grey bars, Group 2a), 225 mg (blue bars, Group 2b) and 375 mg (green bars, Group 2c) bid (mean ± SD). Platelet aggregation was induced by 2 × 10^-5^ M adenosine diphosphate (ADP). No significant inhibition of platelet final aggregation could be measured in LTA (Friedman Test, one-way repeated measures ANOVA). In Group 2c (green bars) values declined just below the cut-off value (red line, 64.2%). **B–C**: Platelet final aggregation of Sheep 1 **(B)** was not inhibited by administration of 150 mg clopidogrel bid. Increasing the dosage up to 225 mg (blue bars Group 2b) or 375 mg (green bars, Group 2c) bid, values on day 7 declined below the cut-off value (red line, 64.2%). By contrast, in Sheep 3 **(C)** an adequate effect of clopidogrel in every dosage can be seen on days 4 and 7 (values below the cut-off value).

Nonetheless, more detailed analysis of the sheep values revealed a trend towards inhibiting of ADP-FA on day 7 with the increment of the concentration (Figure 2A) (150 mg bid: 73.8 ± 28.7%, 225 mg bid: 66.8 ± 29.4%, 375 mg bid: 59.6 ± 24.0%). Values below the cut-off value (64.2%) could be measured on day 7 (ADP-FA: 59.6 ± 24.0%) when administering the highest dosage (375 mg bid) to sheep and 150 mg bid to lambs on days 7 and 28 (ADP-FA: day 7: 51.8 ± 18.6%, day 28: 57.2 ± 27.8%). In lambs between days 7 and 28, no significant difference could be detected.

Considering the sheep and lambs individually in relation to the cut-off values, intersubject variability can be observed. ADP induced platelet FA decreased below the cut-off value in 2 out of 5 sheep and in 3 out of 5 lambs. In Sheep 1, the increase in dosage from 150 mg to 225 mg and 375 mg bid induced an inhibition of ADP induced platelet FA below the cut-off value on day 7 (Figure 2B) (150 mg bid: 76% vs. 225 mg bid: 54% vs. 375 mg bid: 47%). In Sheep 3, 150 mg bid was sufficient to inhibit ADP induced platelet final aggregation below the cut-off value from day 4 on. Raising the dosage to 225 mg and 375 mg bid did not further influence this effect (Figure 2C). Comparing Groups 2c (sheep, 375 mg = 5.36–6.25 mg/kg bid) and 2d (lambs, 150 mg = 5.0–6.0 mg/kg bid), almost receiving the same dosage per kilogram, no significant difference could be measured.

BT did not change significantly in each of the concentrations tested.

Clopidogrel and clopidogrel carboxylic acid were detected in every plasma sample. An analysis of the concentrations of clopidogrel and its main inactive metabolite clopidogrel carboxylic acid was performed in Groups 2c (sheep, 375 mg = 5.36–6.25 mg/kg bid) and 2d (lambs, 150 mg = 5.0–6.0 mg/kg bid) on baseline, days 1, 4, 7 and 28 (lambs), respectively.

**Figure 3:**
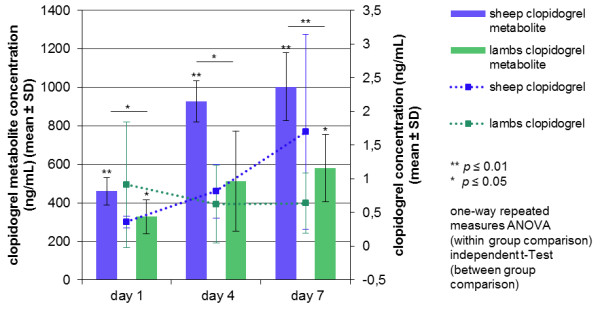
**Clopidogrel and clopidogrel metabolite could be detected in every plasma sample in sheep and lambs.** Both clopidogrel and its main inactive metabolite clopidogrel carboxylic acid could be measured in each plasma sample during the 7-day treatment with 375 mg (5.36–6.25 mg/kg, sheep) and 150 mg (5.0–6.0 mg/kg, lambs) bid. A significant and highly significant increase in the metabolite plasma concentration between baseline and measurement time points in sheep (blue bars) and in lambs (green bars) could be observed (one-way repeated measures ANOVA). There were significant and highly significant differences concerning the metabolite concentration between sheep and lambs (day 1: *p* = 0.031, day 4: *p* = 0.011, day 7: *p* = 0.005, independent t-Test, post hoc Bonferroni Test). The animal's age had a highly significant influence on the concentration of the metabolite (*p* = 0.004, two-way repeated measures ANOVA). In sheep and lambs, clopidogrel could be detected in each sample on days 1, 4, 7 and 28, respectively. However, there was no significant difference between the measurement time points in sheep (one-way repeated measures ANOVA) and in lambs (Friedman Test, post hoc Wilcoxon Signed Rank Test). Values among sheep and lambs differed markedly. No significant differences in clopidogrel plasma concentration could be detected between sheep and lambs (independent t-Test, Mann–Whitney U-Test).

During the 7-day treatment with 375 mg (sheep) and the 28-day treatment with 150 mg (lambs) clopidogrel bid, clopidogrel and its metabolite clopidogrel carboxylic acid were measured in each sample from all sheep and all lambs (Figure 3).

Calibration range was 0.2–3.6 ng/mL for clopidogrel and 100–3600 ng/mL for clopidogrel carboxylic acid. Intraday coefficients of variation ranged from 2.1 to 7.5% for clopidogrel and from 2.3 to 3.7% for clopidogrel carboxylic acid. The intraday accuracies ranged from 94.6 to 99.1% for clopidogrel and from 100.0 to 107.9% for clopidogrel carboxylic acid.

There was a highly significant difference in concentration of clopidogrel carboxylic acid between the measurement time points in sheep (*p* ≤ 0.001) and in lambs (*p* = 0.005). In sheep, highly significant differences were measured between every time point (baseline–day 1: *p* = 0.001, baseline–day 4: *p* ≤ 0.001, baseline–day 7: *p* = 0.001, day 1–4: *p* = 0.006, day 1–7: *p* = 0.009), with the exception of days 4 and 7. In lambs, a significant increase from baseline to day 1 (*p* = 0.011), day 7 (*p* = 0.017) and day 28 (*p* = 0.024) could be detected. No significant change was seen between all other measurement time points.

In sheep and lambs, clopidogrel could be detected in each sample on days 1, 4, 7 and 28, respectively. Values among sheep and lambs differed greatly. There were no significant differences between the measurement time points in sheep and lambs.

There were significant and highly significant differences concerning the clopidogrel carboxylic acid concentration between sheep and lambs on days 1, 4 and 7 (day 1: *p* = 0.031, day 4: *p* = 0.011, day 7: *p* = 0.005). The animal's age had a highly significant influence on the concentration of the metabolite (*p* = 0.004).

No significant differences in clopidogrel plasma concentration could be detected between sheep and lambs.

There was no association noticed between clopidogrel or clopidogrel carboxylic acid concentration and ADP-FA levels in the individual sheep.

### Ticagrelor inhibited platelet aggregation in 1 out of 5 sheep

In Group 3, the antiplatelet agent ticagrelor was administered to sheep in four different dosages (Group 3a: 90 mg bid, Group 3b: 180 mg, Group 3c: 360 mg bid, Group 3d: 540 mg bid) for a time period of 7 days. The efficacy was tested in LTA and BT on baseline, days 1, 4 and 7.

**Figure 4:**
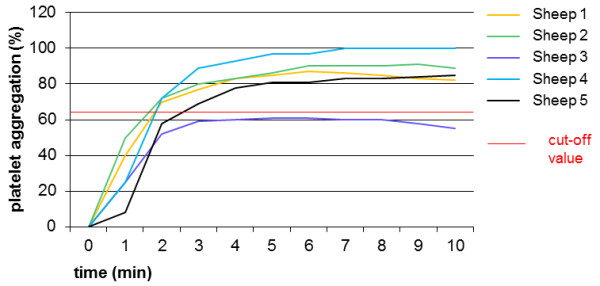
**Light transmission aggregometry after administration of ticagrelor (Group 3d).** Platelet aggregation was induced by adenosine diphosphate in a concentration of 2 × 10^-5^ M. Regarding the platelet final aggregation (time point 10 minutes) on day 7 with ticagrelor in a dosage of 540 mg bid, an inhibition below the cut-off value (red line, 64.2%) could only be noticed in Sheep 3 (final aggregation: 55%). The other sheep showed no response, with line profiles almost equal and platelet aggregation values ranging from 82–98%. Calculating the results of all sheep, no significant differences could be noticed between baseline, days 1, 4 or 7 (one-way repeated measures ANOVA).

Mean values after administration of ticagrelor showed no significant change in platelet final aggregation between the measurement time points. ADP induced platelet final aggregation did not decrease below the cut-off values at any time point. Regarding the values of the individual sheep in Groups 3c and d (Figure 4), an inhibition of ADP induced platelet FA below the cut-off value could only be noticed in Sheep 3 (Group 3c, day 7: 47%; Group 3d, day 7: 55%). There was no significant change between baseline and measurement time points for BT.

### In sheep, human therapeutic anti-FXa levels were achieved using sodium enoxaparin

The FXa inhibitor sodium enoxaparin was administered to sheep in two different dosages over 7 days. Its efficacy was evaluated through anti-FX activity in plasma on baseline, days 1, 4 and 7.

**Figure 5:**
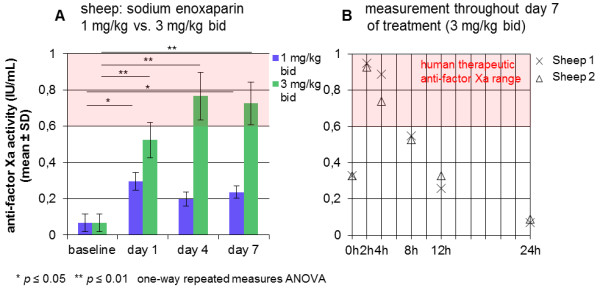
**Human therapeutic anti-factor Xa levels in sheep after administration of sodium enoxaparin (Groups 4a–b). A**: After the administration of sodium enoxaparin in a concentration of 1 mg/kg bid (loading dose: 2 mg/kg, Group 4a), human therapeutic anti-factor Xa levels (0.6–1.0 IU/mL) could not be achieved (blue bars). There was a significant increase in anti-FXa levels comparing reference values with day 1 (*p* = 0.027) and 7 (*p* = 0.028). Between baseline and day 4 values did not differ significantly (one-way repeated measures ANOVA, post hoc Bonferroni Test). The administration of 3 mg/kg sodium enoxaparin (Group 4b) induced values within the human therapeutic range from day 4 on (green bars). A highly significant increase was seen between reference values and day 1 (*p* = 0.007), day 4 (*p* = 0.005) and 7 (*p* = 0.003), respectively. Between days 4 and 7, values did not differ significantly, indicating the formation of a steady state (one-way repeated measures ANOVA, post hoc Bonferroni Test). **B**: Measurement of the anti-factor Xa concentration throughout day 7 of treatment with 3 mg/kg sodium enoxaparin bid of 2 sheep. Peak plasma levels (0.94 ± 0.01 IU/mL) could be detected 2 hours after the administration of 3 mg/kg. Values rapidly decreased in the following hours to finally 0.08 ± 0.01 IU/mL 24 hours after injection.

The administration of a loading dose of 2 mg/kg and 1 mg/kg bid subcutaneous (s.c.) in Group 4a induced anti-FXa levels below the human prophylactic range (0.40–0.60 IU/mL) (day 1: 0.30 ± 0.05 IU/mL, day 4: 0.20 ± 0.04 IU/mL, day 7: 0.24 ± 0.03 IU/mL). There was a significant increase in anti-FXa levels, comparing reference values (0.07 ± 0.05 IU/mL) with day 1 (*p* = 0.027) and 7 (*p* = 0.028). Between baseline and day 4, values did not differ significantly (Figure 5A, blue bars).

In Group 4b (3 mg/kg bid s.c.) values within the human therapeutic range (0.60–1.00 IU/mL) were measured from day 4 on. A highly significant increase was seen between reference values and day 1 (0.52 ± 0.10 IU/mL, *p* = 0.007), day 4 (0.77 ± 0.13 IU/mL, *p* = 0.005) and day 7 (0.73 ± 0.12 IU/mL, *p* = 0.003), respectively. Between days 4 and 7, values did not differ significantly (Figure 5A, green bars).

Exemplary measurements over 24 hours on day 7 of 2 sheep in Group 4b were performed. The anti-FXa activity throughout 24 hours revealed peak plasma levels (0.94 ± 0.01 IU/mL) 2 hours after administration of 3 mg/kg, which rapidly decreased in the next hours to finally 0.08 ± 0.01 IU/mL 24 hours after injection (Figure 5B).

### In lambs, human therapeutic anti-FXa levels were achieved using sodium enoxaparin in a higher dosage than in sheep

The anti-FXa inhibitor sodium enoxaparin was administered to lambs in two different concentrations over 7 days. Its efficacy was evaluated through anti-FX activity in plasma on baseline, days 1, 4 and 7.

**Figure 6:**
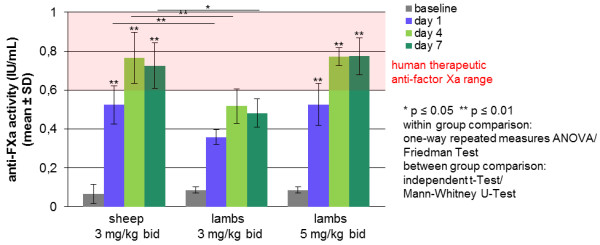
**Anti-factor Xa activity of lambs compared to sheep after administration of sodium enoxaparin.** Highly significant differences in the anti-FXa activity were observed administering the same concentration (3 mg/kg bid) of sodium enoxaparin to lambs (Group 4c) and to sheep (Group 4b) (day 1: *p* = 0.008 independent t-Test, day 4: *p* = 0.008 independent t-Test, day 7: *p* = 0.016 Mann–Whitney U-Test). The treatment of lambs with 3 mg/kg bid induced no significant increase between reference values and days 1, 4 and 7 (Friedman Test, post hoc Wilcoxon Signed Rank Test). However, anti-factor Xa concentrations in the human therapeutic range (0.60–1.0 IU/mL) could be reached administering 5 mg/kg sodium enoxaparin bid to lambs. These data are comparable to Group 4b (sheep, 3 mg/kg bid) without any significant differences (two-way repeated measures ANOVA). In these two groups, there was a highly significant increase comparing reference values with days 1, 4 and 7, respectively (*p* ≤ 0.01, one-way repeated measures ANOVA).

The administration of 3 mg/kg s.c. bid to lambs in Group 4c could not achieve human therapeutic anti-FXa activity (0.6–1.0 IU/mL) (day 1: 0.36 ± 0.04 IU/mL, day 4: 0.52 ± 0.09 IU/mL, day 7: 0.48 ± 0.07 IU/mL). No significant increase between reference values (0.09 ± 0.02 IU/mL) and days 1, 4 and 7 could be seen (Figure 6).

Comparing these results (Group 4c, lambs 3 mg/kg bid) with sheep (group 4b, 3 mg/kg bid) receiving the same concentration of sodium enoxaparin per kilogram, significant and highly significant differences in the anti-FXa activity were observed (day 1: *p* = 0.008, day 4: *p* = 0.008, day 7: *p* = 0.016), revealing the age to have a significant influence on the effectiveness of sodium enoxaparin (Figure 6).

Raising the dosage to 5 mg/kg s.c. bid in lambs in Group 4d resulted in anti-FXa activities in the therapeutic range from day 4 on (day 1: 0.53 ± 0.11 IU/mL, day 4: 0.77 ± 0.04 IU/mL, day 7: 0.77 ± 0.10 IU/mL). There was a highly significant increase between reference values and days 1 (*p* = 0.004), 4 (*p* ≤ 0.001) and 7 (*p* = 0.001). Between days 4 and 7, values did not differ significantly (Figure 6).

Therapeutic anti-FXa activity was achieved with the high dosage in lambs (Group 4d, 5 mg/kg bid) on days 4 and 7 and differs not significantly from that in sheep with the high dosage (Group 4b, sheep, 3 mg/kg bid) (Figure 6).

### Interindividual different and delayed response to dabigatran etexilate

The direct thrombin inhibitor dabigatran etexilate was administered to sheep over 7 days in a dosage of 600 mg bid p.o. Its efficacy was evaluated using the Hemoclot® test.

**Figure 7:**
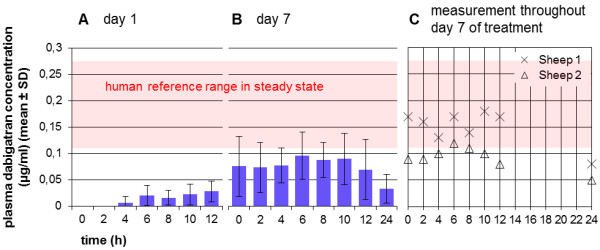
**Interindividual different and delayed response to dabigatran etexilate (Group 5).** Sheep were treated with 600 mg dabigatran etexilate for 7 days. **A**: Dabigatran could be detected in plasma 4–8 hours after the first oral application of 600 mg dabigatran. Highly individual differences between sheep were noticed. **B**: Both on days 4 and 7, maximum dabigatran concentrations were measured at a time point of 6 hours. Twenty-four hours after the last administration, dabigatran plasma concentration was 0.034 ± 0.027 μg/mL. There were no significant differences when comparing reference values (0.000 μg/mL) and maximum dabigatran plasma levels on days 1, 4 and 7 (one-way repeated measures ANOVA, post hoc Bonferroni Test). **C**: Interindividual differences could be noted when considering values of the single sheep at the different measurement time points. Only 2 sheep reached plasma concentrations of dabigatran in the range of human values on day 7.

In plasma, dabigatran could be determined 4–8 hours after the first oral application on day 1 (Figure 7A). On days 4 and 7, maximum dabigatran concentrations were measured at a time point 6 hours after application (day 7, Figure 7B). Measurements 24 hours after the last application resulted in dabigatran plasma concentration of 0.034 ± 0.027 μg/mL (Figure 7B). There were no significant differences when comparing reference values (0.000 μg/mL) and maximum plasma dabigatran plasma levels on day 1, day 4 and day 7.

Interindividual differences could be noted when considering values of the individual sheep on the different measurement time points. In 2 sheep, plasma concentrations of dabigatran in the range of human values were detected on day 7 (0.117–0.275 μg/mL, mean 0.175 μg/mL, 2 hours after administration of 150 mg bid in the steady state) (Figure 7C).

## Discussion

In this study, the efficacy of different antiplatelet drugs and anticoagulants was evaluated in sheep of different ages in order to establish a reliable antiplatelet and anticoagulation therapy for sheep in experimental research. Two different aggregometry systems were assessed for their ability to measure platelet function in this species, because to date no standardised method has been described for the evaluation of platelet aggregation in sheep.

### Light transmission aggregometry, but not impedance aggregometry, serves as a reliable testing system for evaluation of reference values in healthy and untreated sheep

The impedance aggregometry provides several advantages compared to LTA, for example, the usage of whole blood samples preserving the natural environment of the platelets and the elimination of the time-consuming centrifugation step, thus avoiding the collection of large blood volumes and the loss of large platelets. The platelet volume significantly affects its function [20] with the mean platelet volume correlating with platelet function and activation [21]. However, this system has essentially proved unsuitable for the evaluation of platelet function in sheep. Our data are concordant with those of the study of Baumgarten et al., in which neither AA nor TRAP, could induce measurable platelet aggregation in healthy sheep [22]. One can assume that there might be other potent ways for the induction of platelet aggregation in sheep than stimulation by AA. The lack of response to TRAP was already regarded by Baumgarten et al. as a reflection of a species-specific thrombin receptor. Therefore it was not considered essential to test this system with collagen, because the same testing system should be used for all groups in the study. In the LTA, still considered the gold standard for evaluation of platelet function [23], platelet aggregation could be induced with all agonists needed for the study (ADP, collagen and AA). For this reason this system was used in every single group.

In every group blood was anticoagulated with citrate according to the Recommendations of the International Society on Thrombosis and Haemostasis [24]. However, it would be possible to anticoagulate the blood with other agents, such as hirudin or to use washed platelets to reduce a possible effect of the low calcium concentration in PRP of citrate anticoagulated blood [25].

The recommended concentrations of ADP and collagen for human PRP achieved sufficient platelet aggregation in sheep PRP. AA concentration had to be increased up to 10.93 mmol/L. Sheep possess a higher number of platelets in contrast to humans and therefore possibly need a higher concentration of agonists for the induction of platelet aggregation. There seem to be some fundamental differences in platelet aggregation processes of sheep compared to humans. Goodman et al. examined platelet-material interactions and showed that platelets of sheep attached to surfaces much less, never reached fully spread shapes and displayed a lower overall activity than human platelets [12]. The adhesion of sheep platelets to immobilised fibrinogen surfaces was, in contrast to other animals, only possible when stimulated by ADP, although to a lesser extent than platelets from other animal species [26].

The results of AA induced aggregation in this study are comparable with previously recorded data showing that low (2 mmol/L [27]/ 0.5 mmol/L [28]) compared to high (7.5 mmol/L [11]) concentrations could not reach complete sheep platelet aggregation in the LTA. In this study, a concentration of 10.93 mmol/L AA was identified to be crucial for an adequate level of aggregation. This difference could be due to a 3–4 fold interindividual variation, as already mentioned by Baumgarten et al. [22], especially when working with small numbers of animals. In addition, the agonists are obtained by different manufacturers and therefore could vary in their biological activity [22].

In our laboratory calculated human cut-off values for the different agonists are established for clinical routine use, however not for sheep. These reference values should not be used due to the differences between human and sheep platelet aggregation and anticoagulation systems. To increase the reliability of the results of the study, new cut-offs with blood from healthy untreated sheep of the same breed were calculated, which revealed quite small interindividual differences. For data comparison in every individual sheep, baseline values were measured before starting the medication.

### ASA could not inhibit sheep platelet aggregation both *in vivo* and *in vitro*

There are still many research groups administering acetylsalicylic acid (ASA) for the prevention of thromboembolic events in sheep, without knowing if there is any antiplatelet effect [29]. ASA induces an inhibition of platelet function by irreversible inactivation of the cyclooxygenase activity of prostaglandin H-synthase-1 [30], also known as cyclooxygenase-1 (COX-1). In our study, inhibition of platelet aggregation through the application of ASA could be achieved neither *in vivo* nor *in vitro*. Data have been published about the effect of ASA and its pharmacokinetics in sheep, but never examining the antiplatelet effect of ASA. In a previous study, the absorption process of salicylate after oral administration seemed particularly inefficient in the ruminant animal [31], in this case possibly due to the coating with gelatin.

The high pH of the rumen favours the existence of the ionised and non-diffusible form of ASA. In contrast, the non-ionised and diffusible form dominates in the stomach of monogastrics. Nevertheless, in cattle bioavailability of 70% could be achieved with high oral salicylate concentrations [32].

Therefore, the ineffectiveness of the drug is most likely not caused by insufficient absorption. When given intravenously, a rapid elimination of salicylate from the plasma of cattle, particularly of goats and sheep, was demonstrated, probably on the basis of high renal excretion rates of salicylate [31,32]. ASA induced antiplatelet effects are attained with only low concentrations and persist over some days, even when ASA already has been eliminated from plasma due to the irreversible effect on platelets. Thus, the rapid disappearance after venous administration is probably not the explanation for the ineffectiveness.

When examining the effect in the *in vitro* experiment, no inhibition of platelet aggregation could be measured. In the positive control (human PRP), AA induced platelet aggregation was inhibited with ASA sufficiently, however not the collagen induced platelet aggregation. Collagen is probably not as suitable as an agonist, because inhibition of an aggregation response by aspirin occurs at lower inhibition rates of thromboxane A_2_ (TXA_2_) synthesis and it is more variable. AA is described as a more suitable platelet agonist than others (for example ADP, collagen), which are working through pathways that are less dependent on thromboxane production [33]. To prove a sufficient effect of aspirin on platelet aggregation a nearly complete inhibition of TXA_2_ synthesis would be a necessary prerequisite to prevent aggregation induced by a strong agonist such as thrombin and could be therefore a system to test for aspirin resistance. Furthermore one has to keep in mind, that there are different types of collagen which can lead to variance in their reactivity. For example collagen type I, II, III, IV are described to be more reactive than other types (VI, VII, VIII) [34]. In another study collagen type III was found to be a more potent inducer of platelet aggregation than type I and II [35]. Possibly we could achieve different results using other types of collagens. Moreover collagen concentration was quite high in our experiments; probably with lower concentrations inhibition of platelet aggregation could be detected.

The experimental setup was similar to an early study of Spanos et al. [36], with the difference of using other concentrations of aggregation inducing agonists and a higher concentration of ASA in accordance to the ASA control of the Multiplate® analyser (1 mg/mL).

Moreover, two previous studies have shown a 37- and 31-fold better ASA induced inhibition of prostaglandin synthetase from human platelets than from sheep seminal vesicles, unclear whether depending on the different tissue origin or not [37,38]. Finally, it can be assumed that sheep platelet aggregation occurs to a large extent independently of AA and TXA_2_, as has already been proposed [36]. The need of very high concentrations of AA in the LTA to induce aggregation supports this hypothesis.

On the other hand, there could be a TXA_2_ production despite ASA administration, either by insufficient inhibition of COX-1 or COX-1 independent production of TXA_2_. Measurement of TXB_2_ in blood serum could serve for an indirect assessment of the concentration of TXA_2_[39]. It should be an issue of future studies to determine the underlying reasons for the ineffectiveness of ASA in sheep.

### Clopidogrel inhibited ADP induced platelet final aggregation adequately in 2 out of 5 sheep and in 3 out of 5 lambs, while clopidogrel and clopidogrel carboxylic acid were detected in every plasma sample

Clopidogrel is an oral ADP receptor antagonist which inhibits platelet activation through blockade of the P2Y_12_ ADP receptor on the platelet surface [40]. An inhibition of sheep platelet aggregation just below the cut-off value could be achieved on day 7, when administering the highest dosage. The high standard deviations resulted from the high intersubject variability. In 2 out of 5 sheep and 3 out of 5 lambs an effective inhibition of platelet aggregation could be shown. No significant changes between days 7 and 28 could be seen, thus indicating the formation of a steady state after the one-week treatment. In Sheep 3, sufficient inhibition of platelet aggregation could be reached even with the lowest dosage of clopidogrel, whereas Sheep 1 required a higher dosage for effective inhibition of platelet aggregation. This observation is possibly due to clopidogrel resistance: in humans platelet aggregation of low- or non-responders can be inhibited more effectively when increasing the dosage of clopidogrel [41]. Connell et al. noted only a modest antiplatelet response to clopidogrel, when treating 2 sheep and goats with 75–150 mg/day [42].

After absorption, the main and inactive metabolite clopidogrel carboxylic acid is formed by intestinal or hepatic esterase. Only a very small amount of about 15% is metabolised in the liver through the activity of different isoenzymes of the P450 system. It is well known that there are interspecies differences and variations concerning the drug metabolism mediated by the cytochrome P450 system [43].

One of the most frequently discussed reasons for clopidogrel ineffectiveness is the genetic polymorphism in the cytochrome P450 system (CYP2C19) [44]. CYP2C19 is a highly polymorphic enzyme with more than 25 different alleles. The most common CYP2C19 loss-of-function allele is the allele *2. While homozygous carriers (*2/*2) have a very low metabolism, in heterozygous carriers (*1/*2) it is only reduced [45]. However, there are no data concerning the existence of these gene variants in sheep.

In humans, clopidogrel resistance can occur in the case of inadequate compliance, poor absorption, non-optimal dosages, smoking, gene polymorphisms or drug interactions [46].

However, in sheep, limited efficacy of clopidogrel could be due to a reduced absorption of clopidogrel, a degradation or inactivation in the forestomach system, or an accelerated clearance of the active metabolite, or the above-mentioned genetic variations concerning the cytochrome P450 system or the P2Y_12_ target receptor. Other explanations for the limited clopidogrel effects are possibly a higher number of P2Y_12_ receptors on sheep platelets, other more important ways of the initiation of platelet aggregation, an accelerated platelet turnover, or a higher number of platelets in sheep.

In every sample from all sheep, clopidogrel and the inactive metabolite clopidogrel carboxylic acid were measured. In 3 out of 10 baseline samples clopidogrel was measured. Most likely, this was due to laboratory contamination as definitely none of the animals had received any drugs 2 weeks prior to blood collection. The inactive metabolite clopidogrel carboxylic acid could be detected in concentrations lower than expected for such a high dosage. Levels comparable to those in humans treated with 75 mg/day could not be reached [47]. Besides a poor absorption, other reasons for the low metabolite concentrations are probably a lower conversion of clopidogrel or a higher clearance rate of the metabolite.

An age-dependency of the concentration of clopidogrel carboxylic acid in plasma could be revealed. Clopidogrel carboxylic acid concentrations were significantly lower in lambs, although receiving a comparable dosage of clopidogrel, probably due to an accelerated clearance of the metabolite in young animals. Regarding the individual values of sheep and lambs, the metabolite concentration seems not to be linked with the effectiveness of clopidogrel as an antiplatelet drug. There was no difference in plasma clopidogrel concentration between sheep and lambs. Taken into account the high individual variability and the high conversion rate of another clopidogrel metabolite (clopidogrel glucuronid) into clopidogrel, it is not yet possible to draw a final conclusion on its exact concentration in sheep blood. Lambs were treated with clopidogrel for 28 days to investigate if there was an accumulation or an increase of the clearance of this drug, but neither clopidogrel carboxylic acid nor clopidogrel concentration changed significantly.

In consideration of the clopidogrel results, one can presume that an effectiveness of clopidogrel cannot be ensured on the first day of the treatment when the steady state has not yet been reached, even not after a loading dose of 600 mg. Prospective studies with large groups of animals should be performed to generate sufficient data for the evaluation of the reasons and frequency of non- or low-responders in sheep.

### Ticagrelor inhibited ADP induced platelet final aggregation in only 1 out of 5 sheep

When testing the newly developed direct ADP P2Y_12_ inhibitor ticagrelor in Group 3, 4 out of 5 sheep turned out to be non-responders. Only in Sheep 3, already showing good antiplatelet responses to all dosages of clopidogrel, values below the cut-off value could be detected when administering high dosages of ticagrelor. The outcomes of several human clinical studies comparing clopidogrel and ticagrelor in favour of the latter and its additional ability to prevent vasoconstriction through the inhibition of local P2Y_12_ receptors in the vascular smooth muscle [13], suggested ticagrelor as a good alternative to clopidogrel. Furthermore, ticagrelor has been described as being superior to clopidogrel because of its capacity to be effective without the requirement of hepatic conversion. However, this study revealed only a weak efficacy of ticagrelor in sheep. These data raise doubts about a deficiency in the cytochrome P450 system in sheep being a factor for the insufficient effect of clopidogrel. The reasons for the ineffectiveness of ticagrelor and clopidogrel might be similar, because of a comparable mode of action and comparable antiplatelet responses of the sheep. The poorer efficacy of ticagrelor could be due to the reversible binding and the subsequently faster diffusion of ticagrelor from the P2Y_12_ receptor. Moreover, there is a dependency of inhibition of platelet aggregation and the concentration of the drug in plasma available to occupy receptors [13]. Taking these findings together, one reason for the weak efficacy of ticagrelor can be an accelerated clearance of the drug.

### Bleeding time did not serve as a reliable testing system for the evaluation of platelet aggregation inhibitors in sheep

BT is an *in vivo* test for assessing primary haemostasis, often criticised for its high level of errors due to the wide normal range in bleeding time and limited reproducibility [48]. To guarantee a high reliability, a standardised method was established. The values were considered with respect to the baseline of every individual sheep because of the high intervariability. However, no significant prolonging of BT could be demonstrated. The measurement of antiplatelet effects via BT seems to be unreliable in sheep, even when using a standardised technique.

### In sheep and lambs, human therapeutic anti-FXa levels were achieved using sodium enoxaparin in age-dependent dosages

The administration of 3 mg/kg sodium enoxaparin bid in sheep resulted in anti-FXa activities in the range of human therapeutic values, however not on the first day. It would be conceivable giving a loading dose at the beginning of the treatment. Between days 4 and 7, no significant difference of the anti-Xa activity was detectable, thus indicating the formation of a steady state. Twenty-four hours after the last injection, only a very low anti-FXa activity was measured, hence the administration of 3 mg/kg sodium enoxaparin every 12 hours seems to be necessary. The objective was to achieve human therapeutic anti-FXa activities rather than human prophylactic values to ensure the prevention of thromboembolic events after microvascular surgery.

Only few studies have examined the effect of sodium enoxaparin in sheep, however, never taking into consideration the animal's age. Other measurement time points, different anti-FXa assays and weight of the animals complicate direct comparisons [16,17]. Ao et al. investigated the effect of LMWH on intimal hyperplasia and concluded a daily dosage of 1 mg/kg to be adequate [17]. Since, in the present experiment, no haemorrhagic complications were noticed in any sheep, the higher dosage of 3 mg/kg bid seems to be decisive for the avoidance of thromboembolic events in even very small diameter blood vessels in sheep.

When increasing the dosage for lambs from 3 mg/kg bid up to 5 mg/kg bid, values in the human therapeutic range could be measured, which were consistent with those obtained from sheep with a dosage of 3 mg/kg bid. It became evident that age has a significant influence on the effect of LMWH in sheep, as has already been demonstrated by several studies in humans [49]. Likely explanations for the low effect of sodium enoxaparin in lambs, other young animals or children are a higher volume of distribution, an increased total clearance and lower concentrations of antithrombin related to developmental haemostasis [49]. However, some authors have raised doubts about the different antithrombin concentrations being a dependent factor [50,51]. Age-related differences concerning competitive binding to the endothelium, as well as absorption and clearance of the drug seem to be more important mechanisms [51]. Throughout the present study, human therapeutic ranges of anti-FXa activity were considered as target values. Nevertheless, there are no data available whether sheep require this activity to reach therapeutic success and prophylaxis of thromboembolic events. Even in human medicine, there are no generally accepted regimes available that could serve as evidence-based guidelines for the prevention of thrombosis in microsurgery [52]. The establishment of a reliable prophylaxis of thromboembolic events in human and in sheep microsurgery should be an issue of future studies.

An experimental group with heparin was not performed due to the high variability in response in sheep and the short effect duration leading to a necessary administration every 4 hours [15,53].

### Interindividual different and delayed response to dabigatran etexilate

The administration of the direct thrombin inhibitor dabigatran etexilate resulted in dabigatran plasma concentrations similar to human ranges only in 2 out of 5 sheep, probably due to a low absorption rate of the drug. The drug is incorporated in capsules of tartaric acid to guarantee the formation of a local acid environment which supports the successful absorption of dabigatran etexilate [54]. In sheep, the capsule could be damaged due to the physiological ruminating thus resulting in a decrement of absorption.

The delayed onset of action (maximum plasma levels 6 hours after administration) compared to humans (0.5–2 hours) and the high inter-individual differences can also be justified in the special forestomach system and the always filled rumen of sheep. In humans, when the drug is taken together with a meal, time for reaching maximum plasma levels will be prolonged up to 4 hours.

Sheep received quadruple dosages (600 mg) compared to humans, due to the fact that the administration of 300 mg bid proved to be inefficient in a pilot study. In recent studies in swine [55] and rabbit [56] even higher dosages were used. However, the dosage should not be elevated in this experiment due to the efficacy in 2 sheep and the increased possibility of bleeding complications. Because of the unpredictability of an effect of dabigatran etexilate together with a delayed onset of action, the administration of sodium enoxaparin seems to be superior to dabigatran etexilate as anticoagulant. Nevertheless, there are no data available whether sheep require the same plasma concentrations of dabigatran as humans to achieve an prophylactic effect.

It would be an interesting objective for future studies to test the efficacy of these established dosages of antiplatelet and anticoagulant agents in larger groups of animals in order to obtain more representative data. The antithrombotic effect of the different drugs should then be investigated under “real” surgical conditions. Moreover, it could be worth testing to optimise the efficacy when combining an antiplatelet with an anticoagulant drug.

## Conclusion

For the first time, a standardised method for the assessment of platelet function in sheep as well as baseline values were established in this study since no standardised tests have been described so far. The efficacy of different therapeutic strategies for antiplatelet and anticoagulant management in different concentrations was compared in sheep and lambs. In summary, high dosages of clopidogrel or ticagrelor are useful to inhibit platelet aggregation in some sheep, while treatment with ASA cannot reach an antiplatelet response. Examination of the effectiveness of these antiplatelet drugs should always be performed by LTA due to the presence of non- or low-responders among sheep and thus the need for a dose escalation. For a safe anticoagulation regime in sheep with sodium enoxaparin, it should be administered twice daily in age-dependent dosages. When using dabigatran etexilate as anticoagulant, its efficacy should be proven in each individual sheep before starting an experiment to have the possibility of a dose escalation. Moreover, its delayed onset of action has to be carefully considered.

Although only examining a small number of animals, the data provide significant information about widely used and novel antiplatelet and anticoagulant agents in sheep.

The results may contribute to overcoming the problems of high numbers of dropouts due to thromboembolic events and can support the improvement of a safe and reliable thromboembolic prophylaxis in sheep in experimental research.

The results of this study could make a decisive contribution towards the reduction of unnecessary high animal numbers in experimental *in vivo* research.

## Methods

### Experimental model

The animal care committee of the University of Erlangen-Nürnberg and the government of Mittelfranken, Germany approved all experiments (Az. 54–2532.1-44/11, Az. 54.2532.2-2/11). German regulations for the care and use of laboratory animals were observed at all times. The animals were housed in the veterinary care facility under standardised conditions as described previously [57]. 5 female merino landsheep, aged 1½ years, with a body weight of 60–70 kg were used. In every group, the same 5 animals were treated for 7 days with antiplatelet drugs (ASA, clopidogrel, ticagrelor) or anticoagulants (sodium enoxaparin, dabigatran etexilate).

The effect of clopidogrel (Group 2d) and sodium enoxaparin (Group 4c–d) was investigated in the same 5 female lambs, aged 4–5 months, with a body weight of 25–30 kg.

Table 1 summarises the different drugs and dosages.

**Table 1:** Experimental groups

**Group**		**Age**		**Dose**	**Loading dose**	**Testing system**
		**Sheep**	**Lambs**	**(bid)**		
		**n=**	**n=**			
1	a	5		500 mg p.o.		LTA / BT
				(7.14–8.33 mg/kg)		
	b	5		500 mg i.v.		
				(7.14–8.33 mg/kg)		
	c	3		1 mg/mL 10 mg/mL		*in vitro*
2	a	5		150 mg	300 mg	LTA / BT
				(2.14–2.5 mg/kg)	(4.29–5.0 mg/kg)	
	b	5		225 mg	450 mg	
				(3.21–3.75 mg/kg)	(6.43–7.5 mg/kg)	
	c	5		375 mg	600 mg	LTA / BT / clopidogrel / metabolite concentration
				(5.36–6.25 mg/kg)	(8.57–10.0 mg/kg)	
	d		5	150 mg	300 mg	
				(5.0–6.0 mg/kg)	(10.0–12.0 mg/kg)	
3	a	5		90 mg	180 mg	LTA / BT
				(1.29–1.5 mg/kg)	(2.57–3.0 mg/kg)	
	b	5		180 mg	360 mg	
				(2.57–3.0 mg/kg)	(5.14–6.0 mg/kg)	
	c	5		360 mg	540 mg	
				(5.14–6.0 mg/kg)	(7.71–9.0 mg/kg)	
	d	5		540 mg	720 mg	
				(7.71–9.0 mg/kg)	(10.29–12.0 mg/kg)	
4	a	5		1 mg/kg	2 mg/kg	anti-factor Xa activity
	b	5		3 mg/kg		
	c		5	3 mg/kg		
	d		5	5 mg/kg		
5		5		600 mg		thrombin inhibitor test
				(8.57–10.0 mg/kg)		

(BT = bleeding time, LTA = light transmission aggregometry, Group 1: acetylsalicylic acid, Group 2: clopidogrel, Group 3: ticagrelor, Group 4: sodium enoxaparin, Group 5: dabigatran etexilate).

To establish a reliable test system for the verification of sheep platelet aggregation and to calculate reference and cut-off values, blood samples of 20 adult healthy and untreated female merino landsheep were tested.

In every group baseline values were measured on day 0 before receiving the drugs.

### Antiplatelet drugs: ASA, clopidogrel and ticagrelor

Group 1 (n = 5) was treated with 500 mg ASA bid p.o. (Godamed® 500, Dr. R. Pfleger Chemische Fabrik GmbH, Bamberg, Germany) (Group 1a) or i.v. (Aspirin® i.v. 500 mg, Bayer Vital GmbH, Leverkusen, Germany) (Group 1b) via the Vena auricularis. In Groups 2a–c sheep (n = 5) received clopidogrel (Plavix®, 75 mg Filmtabletten, Sanofi-Aventis Deutschland GmbH, Frankfurt am Main, Germany) p.o. in three different dosages: Group 2a: 150 mg bid, Group 2b: 225 mg bid, Group 2c: 375 mg bid. A loading dose of 300 mg (Group 2a), 450 mg (Group 2b) or 600 mg (Group 2c) was administered on the first day.

In Group 2d lambs (n = 5) received clopidogrel in a dosage of 150 mg bid. The loading dose was 300 mg.

Ticagrelor (Brilique® 90 mg Filmtabletten, AstraZeneca GmbH, Wedel, Germany) was given to sheep (n = 5) p.o. in Group 3: Group 3a: 90 mg bid (loading dose 180 mg), Group 3b: 180 mg bid (loading dose 360 mg), Group 3c: 360 mg (loading dose 540 mg), Group 3d: 540 mg (loading dose 720 mg).

During 7-day treatment, the effect of the antiplatelet drugs was investigated using LTA and BT 3 hours after administration on days 1, 4 and 7. In addition, in Group 1b these tests were performed ½ hour after administration of ASA on day 7. In Group 2d, parameters also were tested after a treatment period of 28 days. The tablets were pulverised, dissolved in water and administered p.o. via a syringe.

### Sodium enoxaparin and dabigatran etexilate

The LMWH sodium enoxaparin (Clexane® multidose 100 mg/mL, Sanofi-Aventis GmbH Deutschland, Frankfurt, Germany) was given to sheep (Group 4a: 1 mg/kg s.c. bid, loading dose 2 mg/kg; Group 4b: 3 mg/kg s.c. bid) and lambs (Group 4c: 3 mg/kg s.c. bid; Group 4d: 5 mg/kg s.c. bid) (n = 5) for a time period of 7 days.

The anticoagulant effect was determined measuring the anti-FXa activity on days 1, 4 and 7 4 hours after administration. In addition, in Group 4b the anti-FXa activity was determined after 2, 4, 8, 12 and 24 hours measuring the values of 2 sheep on day 7.

Group 5 (sheep, n = 5) received dabigatran etexilate (Pradaxa® 150 mg Hartkapseln, Boehringer Ingelheim International GmbH, Ingelheim am Rhein, Germany) in a concentration of 600 mg p.o. bid for 7 days. The capsules were given to sheep with concentrate, taking care that the capsules were completely swallowed. Efficacy was evaluated on days 1 (0, 2, 4, 6, 8, 10, 12 hours after administration), 4 (4, 6, 8, 10 hours after administration) and 7 (0, 2, 4, 6, 8, 10, 12, 24 hours after administration).

### Impedance aggregometry

For the assessment of platelet function, through impedance aggregometry, the Multiplate® analyser (Dynabyte Informationssysteme GmbH, Munich, Germany) was used. Chosen agonists were ADP (final concentration 6.5 μM), TRAP-6 (final concentration 32 μM and 320 μM) and AA (final concentration 0.5 mM and 6.5 mM) (all from Dynabyte Informationssysteme GmbH, Munich, Germany). Blood samples were collected by jugular venipuncture with a 14-gauge needle (Introcan Safety®, B. Braun Melsungen AG, Melsungen, Germany). After discarding the first 5 mL, the blood was drawn in 2.6 mL plastic syringes containing hirudin as anticoagulant (Sarstedt Monovette®, r-Hirudin, Nümbrecht, Germany). The samples were left for a rest period of 30 minutes at room temperature. 300 μL of 0.9% sodium chloride, preheated to a temperature of 37°C, were stirred with the same volume of blood for 180 seconds at 37°C. The reaction was started with the addition of 20 μL of the agonist. Aggregation was continuously recorded for 6 minutes.

### Light transmission aggregometry

For testing the antiplatelet effect of the different drugs, the LTA (Born's principle [23]) can be used. After discarding the first 5 mL of the collected blood, it was placed in 10 mL tubes containing 3.13% sodium citrate as anticoagulant (Sarstedt Monovette®, 3.13% Citrat, Nümbrecht, Germany) and left for a rest period of one hour at room temperature. PRP was prepared by centrifuging the blood at 60 × g for 70 minutes, platelet poor plasma (PPP) by centrifuging at 3 200 × g for 20 minutes after removal of the PRP. The platelet count was adjusted to a range of 150 000–250 000/μL by dilution with PPP. Baseline values were evaluated by means of measuring PPP (clear plasma, light transmission set at 100%) and PRP (light transmission set at 0%) in the Platelet Aggregation Profiler® (Model: PAP-8E, Bio/Data Corporation, Pennsylvania, USA). PRP aliquots of 225 μL were incubated at 37°C for one minute, followed by the addition of 25 μL of the different agonists. To maintain platelets in suspension, a magnetic stirrer was added to each sample (stirring rate 1 200 rpm). As agonists, type I calfskin collagen (acid extracted, gallop method) (final concentration 0.19 mg/mL), ADP (final concentration 2 × 10^-5^ M) and AA (final concentration 1.64 mM and 10.93 mM) were used (all from möLab GmbH, Langenfeld, Germany). The extent of platelet aggregation reached after 10 minutes (final aggregation, FA), was quantified photometrically by the calculation of the increase in light transmission.

For the determination of the effectiveness of the antiplatelet drugs, cut-off values were calculated using the results of blood samples of 20 adult untreated, healthy female merino landsheep (cut-off [%] = mean (platelet aggregation) [%] – 2 × SD (platelet aggregation) [%]). Values below the cut-off level indicate the efficacy of the antiplatelet drugs.

### Platelet count

The blood was placed in 2.7 mL tubes containing ethylene diamine tetraacetic acid **(**EDTA) as anticoagulant (Sarstedt Monovette®, EDTA, Nümbrecht, Germany). Platelets were counted manually in a haemocytometer chamber on days 0, 1, 4 and 7 in Groups 1–3.

### Bleeding time

BT was measured at the ventral non-woollen side of the tail base with the sheep in a sitting position. A blood pressure cuff was placed around the base of the tail and inflated to a pressure of 40 mmHg. An incision 5 mm long and 1 mm deep was performed (Surgicutt Adult device; International Technidyne Corporation, Edison, New Jersey, USA). Blood was blotted from the edges of the wound every 30 seconds until bleeding stopped without touching and destroying the clot. The time interval taken for bleeding to stop was noted.

### FXa chromogenic assay for the evaluation of sodium enoxaparin efficacy

After discarding the first 5 mL, blood samples were drawn in 3 mL plastic syringes containing 3.13% sodium citrate as anticoagulant. Anti-FXa activity was measured in plasma prepared by centrifugation at 2 200 × g for 10 minutes.

After diluting 50 μL of the plasma (1:2) (STA Diluent Buffer, Roche Diagnostica Stago S.A.S., Asnières sur Seine, France) and incubation with 100 μL of chromogenic substrate S-2732 for 60 seconds, 125 μL FXa (lyophilised bovine factor Xa) was added, resulting in cleavage of p-nitroaniline (pNA) (all from Coamatic® Heparin, Chromogenix, Milan, Italy). Subsequently, absorbance change was recorded at 405 nm (STA-R Evolution® coagulometer; Roche Diagnostics Deutschland GmbH, Mannheim, Germany).

All standards were prepared from calibration plasma, all controls from control plasma (calibration plasma LMW H 1–3/control plasma LMW H Low and High, Chromogenix, Milan, Italy).

### Hemoclot® Thrombin Inhibitors assay for the evaluation of dabigatran efficacy

Blood collection and plasma preparation were performed as described above for the anti-FXa assay. Dabigatran efficacy was evaluated with Hemoclot® Thrombin Inhibitors assay (Hyphen BioMed, Neuville-sur-Oise, France).

100 μL of normal pooled human plasma and 50 μL of the diluted test plasma (1:8) were incubated in a preheated test tube at 37°C for one minute. Through addition of 100 μL of highly purified human α-thrombin (preheated to 37°C), the clot formation was initiated. Dabigatran concentration in test plasma was directly proportional to the clotting time measured by the STA-R Evolution® coagulometer.

All standards were prepared from calibration plasma, all controls from control plasma (dabigatran calibration plasma 1–3/control plasma, Hyphen BioMed, Neuville-sur-Oise, France).

### Determination of clopidogrel and its metabolite carboxylic acid in sheep plasma

A validated HPLC-MS/MS method (high-performance liquid chromatography with tandem mass spectrometric detection) for the determination of clopidogrel and clopidogrel carboxylic acid, which has already been published for human plasma [47], was used in this study with slight modifications. 100 μL sheep plasma were precipitated by adding 100 μL internal standard solution (clopidogrel-d3 [10 ng/mL in acetonitrile]) and 110 μL acetonitrile (Acetonitrile hypergrade for LC-MS; Clopidogrel-D3 hydrogen sulfate and clopidogrel carboxylic acid, Bertin Pharma, Montigny-le-Bretonneux, France). After centrifugation, the supernatant was transferred to an autosampler vial and 10 μL were injected into the HPLC-MS/MS system (API 4 000 mass spectrometer, Applied Biosystems, Darmstadt, Germany) equipped with an Agilent 1100 HPLC System (Agilent Technologies, Waldbronn, Germany). During all preparation steps, the samples were kept under cooled conditions. The mass transitions and collision energies were m/z 321.9 to 212.2 (23 eV) for clopidogrel, m/z 327.1 to 217.3 (23 eV) for clopidogrel-d3 and m/z 308.2 to 113.1 (59 eV) for clopidogrel carboxylic acid.

### ASA *in vitro*

Blood collection from 3 untreated sheep, PRP and PPP preparation were carried out as described above. ASA solution was prepared freshly through dissolving ASA powder (Aspirin® i.v. 500 mg, Bayer Vital GmbH, Leverkusen, Germany) in aqua ad injectabilia. PRP with a volume of 200 μL was incubated with 25 μL of ASA solution with a final concentration of 1 mg/mL or 10 mg/mL for 3 minutes at 37°C. After addition of 25 μL of the agonist (AA, final concentration 10.93 mM and collagen, final concentration 0.19 mg/mL), aggregometry was started. As control, aqua ad injectabilia instead of ASA solution was used. Reference values were evaluated through adding 25 μL of the agonist to 225 μL PRP. Human plasma served as positive control.

### Statistical analyses

Data are expressed as mean ± standard deviation (SD). Statistical analysis was performed using the statistical package, SPSS 18.0 for Windows (SPSS 18.0, SPSS Inc., IL, USA). Changes of platelet activity, BT, concentration of clopidogrel and its metabolite clopidogrel carboxylic acid, anti-FXa activity and plasma dabigatran concentration (dependent factor) between the different measurement time points (baseline [day 0], days 1, 4, 7 and 28) (independent factor, repeated measure factor) within each group were analysed statistically by one-way analysis of variance (ANOVA) for repeated measures. Bonferroni correction for multiple comparisons was used for between time point comparisons to identify the sources found significant by ANOVA. Normal distribution was confirmed using the Shapiro Wilk Test due to the small group size. The Greenhouse-Geisser correctional adjustment was applied due to the small group size. In the case of no normal distribution, the non-parametric Friedman Test was used together with the post hoc Wilcoxon Signed Rank Test with a Bonferroni corrected significance level (α’ = α/m; α = significance level, m = number of comparisons).

Mean comparisons of anti-FXa activity, clopidogrel and clopidogrel metabolite concentrations between the different age groups were assessed by t-Test. Normal distribution was confirmed as mentioned above. For verification of the homogeneity of variances, Levene's Test of Equality of Error Variances was used. In the case of no normal distribution, the non-parametric Mann-Whitney U-Test was applied with a Bonferroni corrected significance level. In the case of normal distribution, the influence of the age on the different measurement results was analysed statistically by two-way analysis of variance (ANOVA) for repeated measures with age as between-factor and anti-FXa activity as within-factor.

The level of statistical significance was set to *p* ≤ 0.05. A *p*-value ≤ 0.01 was considered to be highly significant.

In addition to the statistical analysis, an evaluation with respect to the established cut-off values was performed in Groups 1–3. In the case of a platelet aggregation below these limits, the effectiveness of the administered drug could be expected.

## Abbreviations

AA: Arachidonic acid; ADP: Adenosine diphosphate; ASA: Acetylsalicylic acid; AUC: Area under the curve; Bid: *Bis in die*, twice daily; BT: Bleeding time; Col: Collagen; EDTA: Ethylene diamine tetraacetic acid; FA: Final aggregation; FXa: Factor Xa; HPLC-MS/MS: High-performance liquid chromatography with tandem mass spectrometric detection; i.v: intravenous; LTA: Light transmission aggregometry; p.o: per os, oral; PPP: Platelet poor plasma; PRP: Platelet rich plasma; s.c: subcutaneous; TRAP-6: Thrombin receptor-activating peptide 6; TXA2: Thromboxane A_2_.

## Competing interests

All authors confirm that there are no conflicts of interest.

This study was funded by the Baxter Innovations GmbH, Vienna, Austria; the Xue Hong and Hans Georg Geis foundation, Nürnberg, Germany; the Staedtler Foundation, Nürnberg, Germany; the research grants from the University of Erlangen-Nürnberg (ELAN program and the Forschungsstiftung Medizin).

## Authors’ contributions

AW, AMB, JPB, REH conceived and designed the experiments. AW, AMB, JR, MM performed experiments. AW, AA, AMB, DK, JPB contributed to data analysis. AW, AA, AMB, JPB, OB, UK contributed to the writing of the manuscript. All authors discussed and commented on the manuscript.

## Authors’ informations

AW: Born on 14 July 1984 in Hamburg, Germany. 2004–2010 studies in Veterinary Medicine at the Ludwig-Maximilians University, Munich, Germany. Since June 2010 Scientific Assistant at the Department of Plastic and Hand Surgery, University Hospital of Erlangen, Germany (chair: Prof. Dr. R. E. Horch) with the main focus on tissue engineering of vascularised bone tissue in the sheep model. 2013 doctoral thesis “Optimization of the generation of axially vascularised and primary stable bone tissue in the sheep arteriovenous loop model for the therapy of critical sized bone defects” (Prof. Dr. R. Köstlin, Center of Clinical Veterinary Medicine, Ludwig-Maximilians University, Munich, Germany). 2013 postdoctoral fellowship, Friedrich-Alexander University of Erlangen-Nürnberg, Germany.

AMB: Born on 17 July 1981 in Malsch, Germany. 2001–2008 studies in Human Medicine at the Albert-Ludwigs University, Freiburg, Germany and the Ruprecht-Karls-University of Heidelberg. 2005–2006 Scientific Assistant at the Tumor Biology Center (chair: Prof. Dr. H. G. Augustin), Freiburg, Germany. 2006–2008 Scientific Assistant at the Department of Vascular Oncology and Metastasis (chair: Prof. Dr. H. G. Augustin), German Cancer Research Center (DKFZ), Heidelberg. 2010 doctoral thesis "Directly auto-transplanted mesenchymal stem cells induce bone formation in a ceramic bone substitute in an ectopic sheep model". Since 2009 Scientific Assistant in the tissue engineering laboratory focusing on bone tissue engineering and angiogenesis research / Fellow at Plastic and Hand Surgery at the Department of Plastic and Hand Surgery, University Hospital of Erlangen, Germany (chair: Prof. Dr. R. E. Horch).

JPB: Born on 1 April 1976 in Hamburg, Germany. 1996–2003 studies in Human Medicine at the Ernst-Moritz-Arndt University, Greifswald, Germany and the Albert-Ludwigs University, Freiburg, Germany. 1999–2004 doctoral thesis "Tissue engineering of skeletal muscle tissue" at the Department of Plastic and Hand Surgery, University Hospital of Freiburg, Germany. 2003–2005 Resident and Assistant at the Department of Plastic and Hand Surgery, Freiburg, Germany (chair: Prof. Dr. G-B. Stark). Since January 2005 Assistant and since 2009 Senior Physician at the Department of Plastic and Hand Surgery, University Hospital of Erlangen, Germany (chair: Prof. Dr. R. E. Horch). November 2007 Science Award by the “Deutschsprachige Arbeitsgemeinschaft für die Mikrochirurgie der peripheren Nerven und Gefäβe” (DAM). November 2010 habilitation "Tissue engineering of vascularised skeletal muscle tissue".
